# Genomic Analysis Reveals Candidate Genes Underlying Sex-Linked Eyelid Coloboma, Feather Color Traits, and Climatic Adaptation in Huoyan Geese

**DOI:** 10.3390/ani13233608

**Published:** 2023-11-22

**Authors:** Junhui Wen, Jincheng Yu, Li Zhang, Haiying Li, Huie Wang, Hongchang Gu, Xiurong Zhao, Xinye Zhang, Xufang Ren, Gang Wang, Anqi Chen, Lujiang Qu

**Affiliations:** 1 Institute of Animal Husbandry and Veterinary Medicine, Beijing Academy of Agricultural and Forestry Sciences, Beijing 100097, China; 2 Department of Animal Genetics and Breeding, National Engineering Laboratory for Animal Breeding, College of Animal Science and Technology, China Agricultural University, Beijing 100193, China; 3 Liaoning Academy of Agricultural Sciences, Shenyang 110161, China; yujincheng_pi@126.com; 4 College of Animal Science, Xinjiang Agricultural University, Urumqi 830000, China; 5 Xinjiang Production & Construction Corps Key Laboratory of Protection and Utilization of Biological Resources in Tarim Basin, Alar 843300, China; 6 College of Animal Science, Tarim University, Alar 843300, China

**Keywords:** goose, eyelid coloboma, feather color, climate adaptation, genome

## Abstract

**Simple Summary:**

Huoyan geese are found in the cold regions of Northern China and have two specific traits. To understand the genetic basis of these traits, i.e., upper eyelid coloboma and gosling feather color, as well as their adaptation to the climate, we used whole-genome resequencing technology to analyze Huoyan geese and local geese from Southern China with different traits. By performing this analysis, we identified candidate genes associated with upper eyelid coloboma and gosling feather color, as well as genes related to climate adaptation in geese. These findings provide valuable insights for the selection of goose breeds.

**Abstract:**

Driven by natural and artificial selection, the domestic Huoyan geese from Northern China have gradually generated specific phenotypes and climatic adaptations. To understand the genetic basis of the two specific phenotypes that are sex linked, including upper eyelid coloboma and gosling feather color, as well as the climatic adaptations of the Huoyan goose, which can contribute to the artificial selection and breeding of geese. We selected Huoyan geese and nine Southern Chinese goose breeds and identified their divergence on the genomic level. Using selective sweep analysis, we found that *PTPRM* on chromosome Z influences the upper eyelid coloboma phenotype of the Huoyan goose, and *TYRP1* is a plausible candidate gene for the Huoyan gosling feather color. We obtained a number of genes related to cold adaptation in Huoyan geese, mainly involved in physiological functions such as metabolism, angiogenesis contraction and circulatory system, apoptosis, immunity, stress, and neural system. The most interesting candidates for cold adaptation are *PIP5K1B* and *NMNAT3* that are associated with energy metabolism and stress. We also obtained some genes related to heat adaptation, including *AGTPBP1*, associated with neurology; *GDA*, associated with skin pigmentation; and *NAA35*, associated with apoptosis. These findings deepen our understanding of the genetics of specific phenotypes and climate adaptation in local geese and provide insights for the selection of goose breeds.

## 1. Introduction

**Figure 1:**
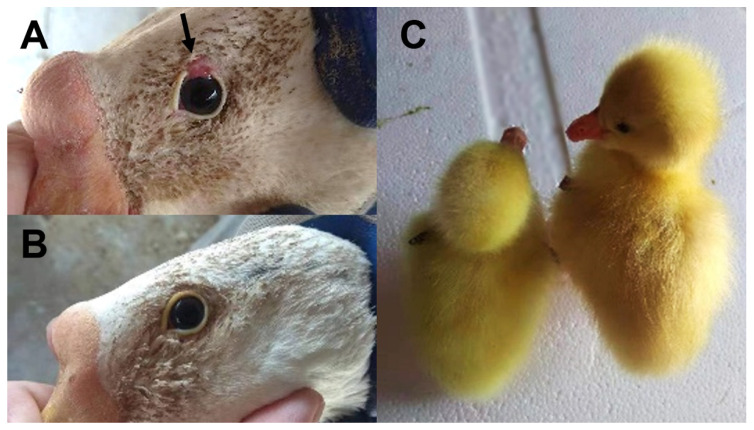
Eyelid coloboma and gosling feather color phenotypes in Huoyan geese. (**A**) Upper eyelid coloboma phenotype of the Huoyan goose marked by the black arrow. (**B**) Normal eyelid phenotype of the Huoyan goose. (**C**) Yellow (left) and light brown (right) feather colors in Huoyan goslings.

The Huoyan goose is one of the valuable local poultry genetic resources in China. The breed is widely dispersed in Liaoning Province in Northern China. Huoyan geese are small-sized with white feathers and orange-colored beaks, tibiae, and flippers. The breed is known for its excellent egg production, with an annual production of up to 120 eggs. Additionally, it has advantageous traits such as feed tolerance, fast growth speed, and high-quality down [1]. One of the most distinctive phenotypic traits of the Huoyan geese is the coloboma of the upper eyelids on both sides, which is present from when they are out of their shells and remains throughout their lives (Figure 1A,B). In 2016, Yu et al. performed cross experiments between Huoyan geese with eyelid coloboma and Sichuan white geese with normal eyelids and found that the eyelid coloboma trait showed a recessive sex-linkage inheritance pattern, and the main locus was located on the Z chromosome [2]. In addition, although the feathers of all adult Huoyan geese are white, there are two down feather color phenotypes in one-day-old goslings, i.e., yellow and light brown (Figure 1C), and the feather color trait of goslings is also sex-linked inheritance [3]. These two unique sex-linked traits in Huoyan geese have significant agricultural value, which can be used for breed identification and gosling sex identification, and serve as an important basis for artificial selection and breeding.

Under long-term natural and artificial selection, the Huoyan geese, which are mainly distributed in Liaoning Province in Northern China, have gradually developed adaptations to cold climates [1]. Liaoning Province is located at 42°35′–43°29′ N and has a cold climate with an average annual temperature of 6.7 °C, reaching as low as −32.5 °C. Therefore, we consider that the Huoyan geese exhibit a certain cold adaptation. China spans about 50 latitudes from north to south, with a wide variety of climate types containing multiple temperature zones. In the lower latitudes of Southern China, where the climate is warmer and more humid, local geese have developed adaptations to heat. At present, genomic selection signals have been widely used to analyze environmental adaptations in domesticated animals, such as the genetic mechanisms of adaptation to high altitude and heat and cold in chickens [4,5]. In 2022, Zhao et al. used genomic and transcriptomic data to reveal the genetic adaptation to cold conditions in chickens [6]. There are no such studies on geese.

In order to explain the unique phenotypes of sex-linked inheritance and climatic adaptations of Huoyan geese, we performed whole-genome resequencing of Huoyan geese and nine other native breeds from Southern China. We identified the Z chromosome of the goose genome and combined three statistical methods (*F*_ST_, θπ ratios, and XP-CLR) to detect the specific phenotypes and cold adaptation of Huoyan geese. Our study enhances the understanding of the specific phenotypes and climatic adaptations of Chinese local geese and provides valuable resources for the breed conservation and hybridization utilization of geese.

## 2. Materials and Methods

### 2.1. Sample Collection

**Figure 2:**
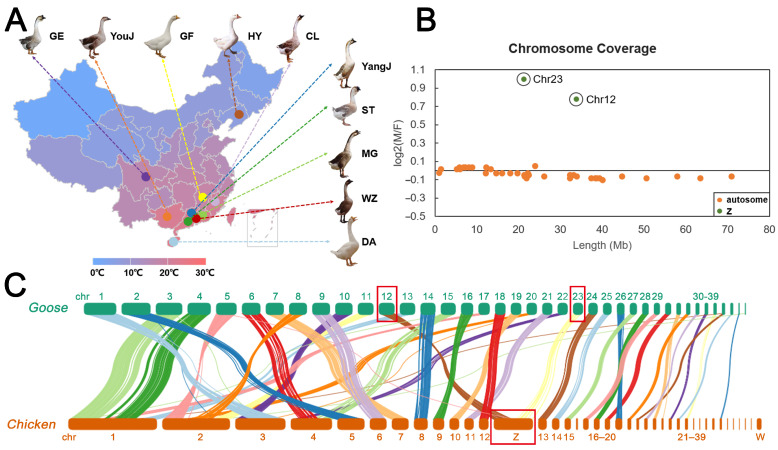
Sample location and Z chromosome identification. (**A**) Location of samples for this study. The color at the bottom indicates the corresponding annual average temperature for each province. (**B**) Male/Female coverage ratios for each chromosome, plotted by chromosome length. Each point represents a single chromosome, and the dark line is the theoretical expectation for autosomes (log2 (M/F coverage) = 0). (**C**) Collinear analysis of goose genome and chicken genome, and the red boxes indicate chromosomes 12 and 23 of the goose and Z chromosome of the chicken.

Whole blood samples were obtained from 72 geese, including 10 native goose breeds from Northern and Southern China (Figure 2A and Table S1). Huoyan geese (HY, *n* = 46) from Liaoning Province in Northern China can be classified into four categories based on phenotypic traits including Huoyan geese with bilateral upper eyelid coloboma (*n* = 38), Huoyan geese with normal eyelid (*n* = 8), one-day-old Huoyan geese with brown down feathers (HYB, *n* = 8), and one-day-old Huoyan geese with yellow down feathers (HYY, *n* = 8). Nine native goose breeds from Southern China (CG) included Dingan goose (DA, *n* = 3), Yangjiang goose (YangJ, *n* = 3), Magang goose (MG, *n* = 3), Lion-head goose (ST, *n* = 3), Wuzong goose (WZ, *n* = 3), Changle goose (CL, *n* = 3), Gang goose (GE, *n* = 2), and Guangfeng white goose (GF, *n* = 3).

### 2.2. Sequencing and Variant Calling

Genomic DNA was extracted using the TIANamp Genomic DNA Kit (DP304-03) (TIANGEN Biotech, Co., Ltd., Beijing, China). The quality and integrity of the DNA were evaluated using the NanoDrop spectrophotometer (Thermo Fisher Scientific Company, Wilmington, DE, USA). Paired-end (150 bp) libraries were prepared and sequenced using the Illumina Novaseq 6000 platform, following the manufacturer’s instructions. To ensure the reliability of the sequencing data, quality control was performed using the NGS QC Toolkit (v2.3.3) with the default parameters [7].

The paired-end reads were mapped to the chromosome-level goose (*Anser cygnoides domesticus*) genome, which was published in the GigaScience database (GigaDB, http://gigadb.org/, accessed on 8 March 2022) [8], using BWA (v0.7.17) [9] with default parameters. The resulting alignments were sorted, and duplicate sequences were removed using Picard [10]. Reads realignment was performed using the RealignerTargetCreator and IndelRealigner tools in GATK (v3.8) [11] to improve alignment accuracy. Variant calling was conducted using UnifiedGenotyper in GATK (v3.6) with a minimum base quality of 20. Variant filtering was executed using VariantFiltration in GATK (v3.6) with the recommended parameters. After the variant filtering step, a total of 17.74 million SNPs were retained and used for subsequent analyses.

Due to the imperfection of the reference genome annotation file, we performed the orthologous protein comparisons of the protein sequences in the reference genome annotation file using the software for eggnog (evolutionary genealogy of genes: Non-supervised Orthologous Groups) [12] to obtain the official names of the protein-coding genes in the reference genome. The result was filtered using the following parameter: --Minimum hit e-value 0.001, --Minimum hit bit-score 60, --Percentage identity 40, --Minimum % of query coverage 20, and --Minimum % of subject coverage 20.

### 2.3. Identification of Sex Chromosomes in Goose

In order to identify the molecular basis of the two Z-linked traits in the geese, the reference genome with Z chromosome is required. However, the reference genome published in 2020 [8] was not annotated with the Z chromosome. For Z chromosome identification, 3 female and 3 male Huoyan goose samples were randomly selected [13], and the median coverage depth of per-base in each chromosome was calculated using the genomecov and groupby in the software BEDtools (v2.30.0) [14], and then, they were normalized by the mean of the median coverages of all chromosomes for each sample. We obtained the mean coverage depth of each chromosome for each sex. Then, we calculated the logarithm of the ratio of male-to-female coverage for each chromosome with a base of 2 (log2 (M/F coverage)) [15]. As the composition of avian female and male sex chromosomes are different, autosomes should have the same coverage depth in different sexes, while Z chromosomes should show approximately a two-fold coverage depth in males.

Then, we performed the whole-genome alignment of the chicken genome (Gallus gallus v7.0) and the goose reference genome using the software NGenomeSyn (v1.41) [16], with parameters set as “-MinLenA 1,000,000, -MinLenB 150,000, -MinAlgLen 8000, -MappingBin minimap2”.

### 2.4. Population Structure Analysis

We performed population structure analyses on the Huoyan geese and nine local goose breeds of Southern China. The software tools VCFtools (v0.1.16) [17] and PLINK (v1.90) [18] were utilized to convert VCF files to PLINK format with stringent filtering parameters: --geno 0.1, --maf 0.1, and --indep-paiwise 20 10 0.5.

After the filtering step, we obtained a total of 7,744,494 variants for the principal component analysis (PCA) using PLINK (v1.90). The PCA analysis was conducted using default parameters to extract the top 20 principal components from the variance-standardized relationship matrix.

The dataset, consisting of 7,744,494 filtered variants, was used to analyze individual admixture and population clustering using ADMIXTURE (v1.3.0) [19]. We converted the VCF file to the ADMIXTURE input file format using PLINK (v1.90). The analysis was performed by assuming a range of ancestral populations, *K*, from 2 to 11. To determine the optimal number of ancestral populations, cross-validation (CV) was implemented using a five-fold value. The *K* value with the lowest CV error represented the optimal number of ancestral populations. The resulting population clustering and individual admixture results were visualized using the Pophelper package [20].

Phylogenetic tree reconstruction was conducted using the SNPhylo [21]. Maximum likelihood (ML) trees were constructed with DNAML programs in PHYLIP (v3.697). The phylogenetic trees were visualized using iTOL [22].

### 2.5. Selective Sweep Analyses

In order to identify candidate regions associated with upper eyelid coloboma traits in the Huoyan goose population, we selected 46 Huoyan geese for selection sweep analysis: 38 samples with upper eyelid coloboma, and 8 samples with normal eyelids. The pairwise nucleotide diversity θπ and genetic differentiation *F*_ST_ [23] were calculated using the software VCFtools, with a sliding window size of 20 kb and a step size of 10 kb. We Z-transformed the *F*_ST_ values. θπ ratios were calculated using the θπ values of the two candidate trait groups, and by log10-transforming the θπ ratios. We considered windows with both top 5% Z(*F*_ST_) and log10 (θπ ratio (normal/coloboma)) as candidate regions that are strongly selected for the upper eyelid coloboma trait. We also estimated the XP-CLR scores [24] between Huoyan geese with normal eyelids and with upper eyelid coloboma, using default parameters, with a sliding window size of 20 kb and a step size of 10 kb, and the threshold was set to the top 1%.

In order to identify candidate regions related to feather color traits in the population of Huoyan geese, 16 Huoyan goslings were selected for the selection sweep analysis, of which eight goslings had a brown feather color and eight had a yellow feather color. We considered windows with both top 1% Z(*F*_ST_), log10 (θπ ratio (brown/yellow)), and XP-CLR scores as candidate regions that are strongly selected for the feather color.

In order to identify the selected regions related to climate adaptations in the Huoyan goose and Southern Chinese local goose populations, we used 72 goose samples, including 46 Huoyan geese from the cold regions of Northern China and 26 local geese from the warm regions of Southern China, for the selection sweep analysis of the Chinese local geese. We considered windows with both top 5% Z(*F*_ST_) and log10 (θπ ratio) as candidate regions that are strongly selected for the climate adaptation. For the θπ ratio (log10 [θπ (HY/CG)]) of the Huoyan geese to the Southern Chinese local geese, the top 5% windows were considered as hot adaption candidates and the bottom 5% were considered as cold adaptation candidates. And we considered windows with top 1% XP-CLR scores as candidate regions strongly selected for the climate adaptation.

The results of the selective sweep analyses were visualized using R (v3.6) [25].

### 2.6. Functional Annotation of Candidate Genes

The intersect function of the software BEDtools (v2.30.0) and custom Python scripts were used to obtain the genes contained in the candidate regions. The common genes within the highly differentiated candidate regions obtained by the three selective sweep methods were considered as candidate genes under strong selection. The obtained gene dataset was functionally annotated using DAVID (v6.8) [26]. The resulting gene ontology (GO) terms were visualized using R (v3.6) [25].

## 3. Results

### 3.1. Identification of Sex Chromosomes in Goose

We conducted whole-genome resequencing on 72 samples for quality control and reference genome mapping. We obtained a total of 776 Gb of high-quality sequences, with an average of 10.79 Gb per individual. Among all the samples, a total of 5173 million mapped reads were obtained, with an average depth of 9.7X and an average coverage of 97.8% per individual (Table S2). After variant calling, we identified a total of 17.74 million SNPs.

To explain sex-linked specific traits in Huoyan geese and to provide the annotation of Z chromosome in the reference genome, we estimated sex-specific patterns of chromosome coverage using resequencing data from female and male Huoyan goose samples. In birds, the sex chromosome is heterogametic (ZW) in female and homogametic (ZZ) in male. Therefore, in males, Z chromosomes should exhibit approximately a two-fold coverage depth compared to females (log2 (M/F coverage) = 1).

After calculation, we observed that all chromosomes, except for chromosomes 12 and 23, showed a similar coverage in both sexes (log2 (M/F coverage) = 0). Chromosome 12 and 23 showed approximately a two-fold coverage in males, consistent with the expectation of Z chromosome (Figure 2B). In addition, the collinear analysis results showed that goose chromosome 12 and chromosome 23 are collinear with chicken Z chromosome (Figure 2C). Therefore, we infer that chromosomes 12 and 23 in the goose genome are parts of the Z chromosome.

### 3.2. Population Genetic Structure

**Figure 3:**
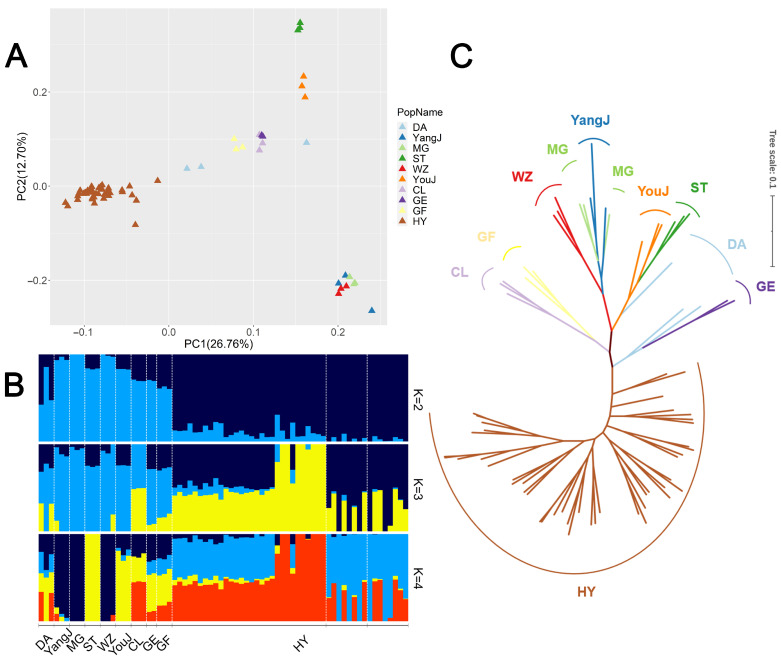
Results of population genetic structure of 72 Chinese local geese. (**A**) PCA plot of goose breeds. (**B**) Population structure of geese. The length of each colored segment represents the proportion of the individual genome from the ancestral populations (*K* = 2–4), and population names are at the bottom. (**C**) ML phylogenetic tree of goose breeds.

The PCA results were visualized using the first two principal components (Figure 3A). In the first principal component, the Huoyan geese showed a separation from other native goose breeds located in the warm areas of Southern China. In the second principal component, there was some clustering among local goose breeds of Southern China. For example, Yangjiang geese (YangJ), Magang geese (MG), and Wuzong geese (WZ) clustered, probably because they are located in similar geographical regions, which are all distributed in Guangdong Province.

The population structure analysis by assigning the number of ancestral populations *K* from 2 to 11 showed the smallest CV error value for *K* = 2 (Table S3). The results are shown in Figure 3B. At *K* = 2 (CV error = 0.65742), Huoyan geese and the Southern Chinese local geese were completely separated. At *K* = 3 and *K* = 4, Huoyan geese and the Southern Chinese local geese still showed obvious differences in genetic components.

To infer the phylogenetic relationships among local goose breeds in Northern and Southern China, we constructed ML trees using genome-wide SNPs. The results showed that the Huoyan geese were separated from the Southern Chinese local geese (Figure 3C).

### 3.3. Selective Signatures of Eyelid Coloboma in Huoyan Geese

**Figure 4:**
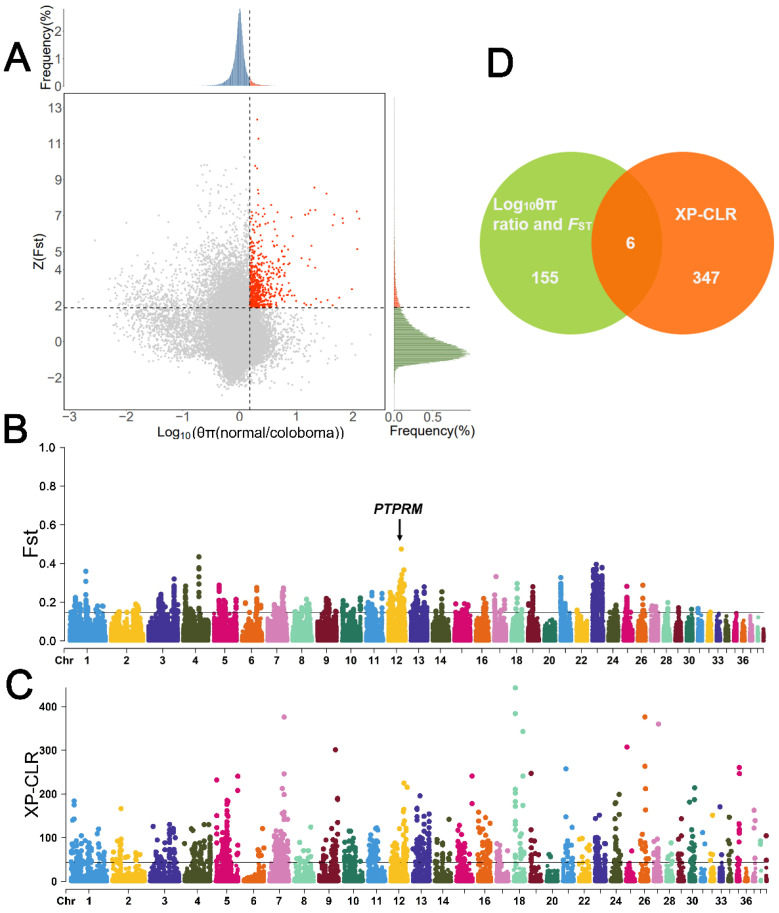
Selective sweep analysis of eyelid coloboma traits in Huoyan geese. (**A**) Results of *F*_ST_ and log10 (θπ ratio [normal/coloboma]) analyses. Horizontal dashed lines indicate the top 5% threshold of Z(*F*_ST_), vertical dashed lines indicate the top 5% threshold of log10 (θπ ratio [normal/coloboma]), and red dots indicate the selected candidate regions for the eyelid coloboma. (**B**) Results of *F*_ST_ analyses. The black line indicates the threshold for the top 1% of *F*_ST_ values. (**C**) Results of XP-CLR analysis. The black line indicates the threshold for the top 1% of XP-CLR score. (**D**) Venn diagram of candidate genes identified using three selective sweep analysis methods.

We calculated *F*_ST_ and θπ values for Huoyan geese with normal eyelids and upper eyelid coloboma using the sliding window method. The top 5% (Z(*F*_ST_) > 1.88, log10 (θπ ratio) > 0.181) windows of the *F*_ST_ and log10 (θπ ratio [θπ normal/θπ coloboma]) results were considered as candidates related to upper eyelid coloboma, and a total of 155 candidate genes were identified (Figure 4A,B). The windows with the top 1% of XP-CLR values (XP-CLR score > 44.1) were obtained for annotation, and a total of 347 candidate genes were identified (Figure 4C). Six shared candidate genes were annotated within the selected regions of the three selection scan methods of *F*_ST_, log10 (θπ ratio), and XP-CLR (Figure 4D, Table S4).

We identified an unstandardized named shared candidate gene evm.model.chr12.1227 (*F*_ST_ = 0.474306, log10 (θπ ratio [normal/coloboma] = 0.311462, XP-CLR score = 158.94938) in the highest peak of the *F*_ST_ value, top 5% of log10 (θπ ratio), and top 1% of XPCLR value on the chromosome 12. The gene CDS sequence was obtained from the reference genome annotation file and using the BLASTN tool of the website NCBI [27], to compare the gene CDS sequence with the database *Anser cygnoides* (taxid:8845). The results of BLSATN comparison showed that the evm.model.chr12.1227 was highly matched with the gene LOC106036398 (receptor-type tyrosine-protein phosphatase mu-like, *PTPRM*) in the goose reference genome (v1.0) [28]. Therefore, our results suggested that *PTPRM*, a gene located on the chromosome 12 that is identified as the Z chromosome, is a strong candidate gene for the upper eyelid coloboma trait.

### 3.4. Selective Signatures of Feather Color in Huoyan Geese

**Figure 5:**
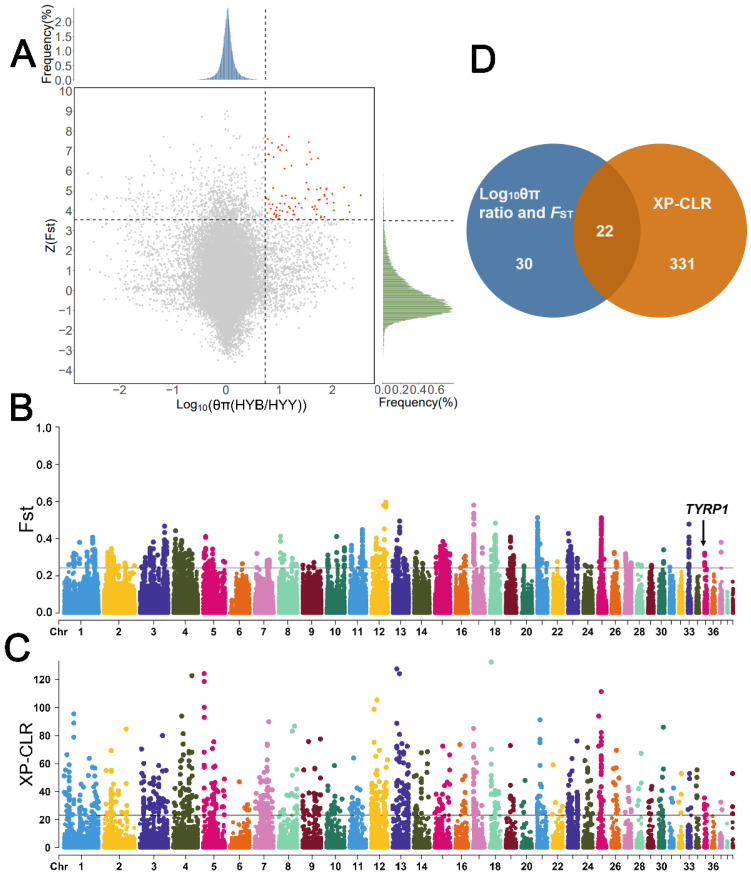
Selective sweep analysis of feather color traits in Huoyan goslings. (**A**) Results of *F*_ST_ and log10 (θπ ratio [HYB/HYY]) analyses. Horizontal dashed lines indicate the top 1% threshold of Z(*F*_ST_), vertical dashed lines indicate the top 1% threshold of log10 (θπ ratio [HYB/HYY]), and red dots indicate the selected candidate regions for the yellow feathers color. (**B**) Results of *F*_ST_ analysis. The black line indicates the threshold for the top 1% of *F*_ST_ values. (**C**) Results of XP-CLR analysis. The black line indicates the threshold for the top 1% of XP-CLR score. (**D**) Venn diagram of candidate genes identified using three selective sweep analysis methods.

We calculated *F*_ST_ and θπ values for Huoyan goslings with brown down feathers and with yellow down feathers using the sliding window method. The top 1% (Z(*F*_ST_) > 3.55, log10 (θπ ratio) > 0.737) windows of the *F*_ST_, and log10 (θπ ratio [HYB/HYY]) results were considered as candidates related to Huoyan goslings feather color, and a total of 30 candidate genes were identified (Figure 5A,B). The windows with the top 1% of XP-CLR values (XP-CLR score > 23.2) were obtained for annotation, and a total of 331 candidate genes were identified (Figure 5C). A total of 22 shared candidate genes were annotated within the selected regions of the three selection scan methods of *F*_ST_, log10 (θπ ratio), and XP-CLR (Figure 5D, Table S5).

Among the shared candidate genes, we identified a tyrosinase-related protein 1 (*TYRP1*) gene in the highest peak of the *F*_ST_ value, top 1% of log10 (θπ ratio), and XP-CLR value on chromosome 35 (*F*_ST_ = 0.321569, log10 (θπ ratio [HYB/HYY]) = 1.3011798, XP-CLR score = 27.444569). *TYRP1* is one of the well-known candidates for feather color-related genes that can lead to the dilution of feather color.

In 2019, Yu et al. found that the feather color of one-day-old Huoyan goose was sex-linked [3]. *TYRP1* is also sex-linked in chickens, quails, and Holdobaggy goslings [29,30,31]. Although the goose reference genome indicates that *TYRP1* is located on chromosome 35, which is considered an autosome (log2(M/F) = 0.012), we conducted further analysis to determine if *TYRP1* is actually located in the sex chromosome region. We compared the male-to-female coverage ratio of *TYRP1* and the plumage color-associated gene *EDNRB2* located on the autosomal (Figure S1). The results showed that the Male/Female coverage ratio in the *TYRP1* gene region was close to 2 (log2 (M/F) = 0.899), which is consistent with the expectation of Z chromosome. In contrast, the Male/Female coverage ratio in the *EDNRB2* gene region was close to 1 (log2 (M/F) = 0.029), which is consistent with the expectation of autosome.

### 3.5. Climate Adaptation of Chinese Local Geese

To explain the cold climatic adaptations of the Huoyan geese, we divided the 72 Chinese local geese into two types based on the latitude of origin of the species. Huoyan geese are cold adaptation geese, and the other nine local goose breeds from Southern China are heat adaptation geese. We calculated *F*_ST_ and θπ values for climate adaptation using the sliding window method.

**Figure 6:**
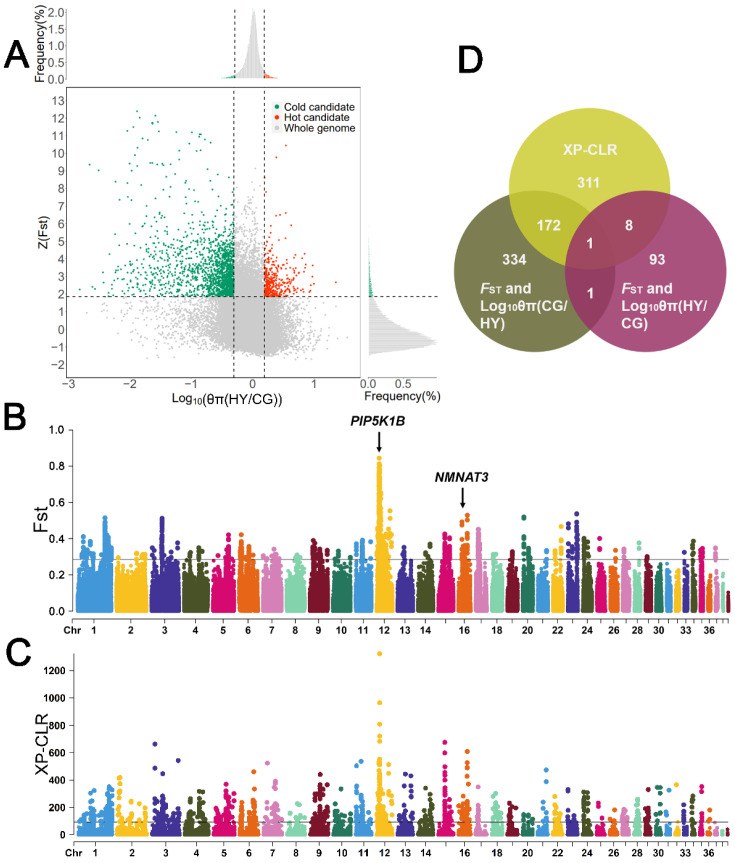
Selective sweep analysis of climate adaptations of Chinese local geese. (**A**) Results of *F*_ST_ and log10 (θπ ratio [HY/CG]) analyses. Horizontal dashed lines indicate the top 5% threshold of Z(*F*_ST_), vertical dashed lines indicate the top 5% threshold of log10 (θπ ratio [HY/CG]), green dots indicate the selected candidate regions for the cold adaptation, and red dots indicate the selected candidate regions for the hot adaptation. (**B**) Results of *F*_ST_ analysis. The black line indicates the threshold for the top 1% of *F*_ST_ values. (**C**) Results of XP-CLR analysis. The black line indicates the threshold for the top 1% of XP-CLR score. (**D**) Venn diagram of candidate genes identified using three selective sweep analysis methods.

The top 5% (Z(*F*_ST_) > 1.86, log10 (θπ ratio [θπ HY/θπ CG]) > 0.192 or < −0.305) windows of the *F*_ST_ and log10 (θπ ratio) results were considered as candidates related to climate adaptation. The top 5% windows of log10 (θπ ratio [θπ HY/θπ CG]) are considered to be related to heat adaptation (log10 (θπ ratio) > 0.192), and the bottom 5% windows are considered to be related to cold adaptation (log10 (θπ ratio) < −0.305) (Figure 6A,B). A total of 425 climate adaptation candidate genes were identified, including 334 cold adaptation candidate genes, 93 heat adaptation candidate genes, and 2 shared candidate genes for cold and heat adaptations. The windows with the top 1% of XP-CLR values (XP-CLR score > 44.1) were obtained for annotation, and a total of 311 candidate genes were identified (Figure 6C), among them there are 173 shared genes for cold adaptation, and 9 shared genes for heat adaptation (Figure 6D, Tables S6 and S7).

Among the cold adaptation candidate genes, we identified two metabolism-related genes (*PIP5K1B* and *NMNAT3*) in the highest peak of the *F*_ST_ value. To further understand the cold adaptation of Huoyan geese, GO functional annotation and KEGG pathway analysis were performed on 173 shared cold adaptation candidate genes, and 70 terms were identified, including 29 significant terms. The results are shown in Figure S2 and Table S8. There are three significant GO terms for biological processes related to cold adaptation, including the regulation of smooth muscle contraction (GO:0006940: *KCNB2*, *PLCE1*, *ADRA2C*), angiogenesis (GO:0001525: *ESM1*, *CLIC4*, *TNFAIP2*, *CYP1B1*, *TSPAN12*, *RBPJ*, *RAMP1*), and apoptotic processes (GO:0006915: *COMP*, *BCL2L13*, *HINT2*, *RALB*, *GRK5*, *API5*, *FAF1*, *MAGI3*, *THOC1*, *BID*, *TGFBR2*). In the term of the regulation of smooth muscle contraction, the gene associated with vasoconstriction is the adrenoceptor alpha 2C gene (*ADRA2C*) [32].

In addition, we also identified a large number of candidate genes related to the regulation of cold-adapted functions, including metabolism-related genes (*ELOVL2* [33], *FASN* [34], *ACOX3* [35], *NDUFS4* [36], and *LPIN1* [37]), heart structure and function-related genes (*KCNK1* [38], *SORBS2* [39], and *NPR2* [40]), immune-related genes (*USP14* [41] and *PI4K2B* [42]), stress-related genes (*VPS13A* [43,44], *NRBF2* [45], *MAP3K21* [46], and *MAPKAPK2* [47]) and neuro-related gene (*SEPSECS* [48]).

Among the nine heat-adapted shared candidate genes obtained from the selection sweep analysis, we identified three candidate genes related to heat adaptation including the neuron-related ATP/GTP binding carboxypeptidase 1 gene (*AGTPBP1*) [49], the metabolism-related guanine deaminase gene (*GDA*), and the apoptosis-related N-alpha-acetyltransferase 35, NatC auxiliary subunit gene (*NAA35*) [50].

## 4. Discussion

In this study, we investigated the characteristic traits and climatic adaptation of the Huoyan geese using whole-genome resequencing data from 48 Huoyan geese and 9 Southern Chinese local goose breeds. Population structure analysis and phylogenetic analysis showed that the Huoyan geese and Southern Chinese local geese were clearly separated into two clusters, which suggested that Huoyan geese and Southern Chinese local geese differed in their genetic material, and their genetic distance was correlated with geographical distance.

### 4.1. Upper Eyelid Coloboma Trait

The results of a selective sweep analysis of the upper eyelid coloboma trait in Huoyan geese showed that the high peaks in *F*_ST_ values were mainly found in chromosomes 12 and 23, and this fact is consistent with the genetic analysis that showed the upper eyelid coloboma trait is a sex-linkage inheritance [2].The candidate gene obtained on chromosome 12 is *PTPRM*, which is a member of the protein tyrosine phosphatase (PTPs) family. PTPs all have at least one catalytic structural domain called PTP structural domain that catalyzes substrate dephosphorylation and plays an important role in many functions such as cell survival, proliferation, differentiation, adhesion, and migration [51]. PTPs can regulate signaling pathways such as receptor tyrosine kinase (RTK) and epidermal growth factor (EGF) [52,53] and can dephosphorylate epidermal growth factor receptor (EGFR) [54]. Decreased *PTPRM* expression leads to EGFR phosphorylation [55]. EGFR is a RTK that regulates the development of craniofacial morphology during embryonic development [56]. It has been shown that EGFR is related to cleft lip and palate and retinal pigment epithelial cell displacement and proliferation [57,58]. Therefore, we suggested that *PTPRM* could be a candidate gene for the upper eyelid coloboma trait. There are few studies related to eyelid coloboma, with reports of candidate genes focusing on *FREM1* [59], which is associated with the phenotypes of anophthalmia, microphthalmia, and upper eyelid coloboma [60]. Interestingly, we identified the *FREM1* gene in the top 1% window of XP-CLR scores of 46 Huoyan geese and in the selected windows of selection sweep analyses of Huoyan geese and Southern Chinese local geese with normal eyelids. Thus, we suggest that the *FREM1* gene on chromosome 12 could also be associated with upper eyelid coloboma.

### 4.2. Feather Color Trait

We identified a candidate gene *TYRP1* associated with Huoyan gosling feather color. And the Male/Female coverage ratio in the *TYRP1* gene region was consistent with the expectation of Z chromosome. *TYRP1* is a member of the tyrosinase gene family, which is specifically expressed in melanocytes and plays a key role in the melanin synthesis pathway in melanosomes [61]. This gene is one of the best-known candidate genes for feather color, and a number of studies have shown that this gene can lead to feather color dilution. In 2007, Nadeau et al. found that a missense mutation located in exon 3 of *TYRP1* was associated with the *roux* phenotype of Japanese quail plumage color [30]. Several studies have shown that chicken chocolate feather color is a sex-linked recessive phenotype associated with missense mutations in *TYRP1* [29,62]. Jiang et al. showed that the down-regulation of genes such as *TYRP1* in a duck’s feather follicles probably contributes to a duck’s white feathers [63]. Xu et al. found that the mRNA expression levels of *TYRP1* genes correlated with the color of the black feathers of the Holdobaggy goslings, and the initial feather color can be used to identify the sex [31]. Therefore, we suggested that the gene *TYRP1* is a plausible candidate gene to control feather color phenotype of Huoyan gosling. In addition, *TYRP1* is located on chromosome 35 in the goose reference genome, which is considered to be an autosomal chromosome. However, the sum of the lengths of chromosomes 12 and 23, which were identified as Z chromosomes by sex chromosome identification, was 55.20 Mb, which was smaller than that of chicken Z chromosome (86.04 Mb) and duck Z chromosome (84.55 Mb); thus, we assumed that Z chromosomes might not be fully assembled and there was an integration of Z chromosome regions into other chromosomes.

### 4.3. Climate Adaptation

Climate adaptation is an important part of the environmental adaptation of domestic animals. It involves a range of physiological responses and relies on the synergy effect of various body organs. Additionally, climate adaptation leads to adaptive changes in the phenotypes and physiological functions of animals. In heat adaptation, animals showed adaptive changes such as reduced basal metabolic rate, vasodilation, hair thinning, enhanced skin pigmentation, and body size changes [64]. In cold adaptation, animals mobilize thermoregulatory mechanisms, including an increased basal metabolic rate and vasoconstriction [65]. In addition, the change in temperature also affects the immune regulation of an animal. In 2015, Kim et al. identified selection signatures such as melanogenesis, body size and development, energy and digestive metabolism, and nervous and autoimmune response in goats and sheep living in hot arid environments [66]. In studies on the rapid adaptation of sheep to extreme environments, the environmental adaptation has shown to affect body size, energy metabolism, and stress response in sheep [67]. In studies on the adaptation of camel to high-heat desert environments, adaptive physiological mechanisms such as lipid metabolism, DNA damage and repair, immune regulation, and osmoregulation were revealed [68]. In 2020, Tian et al. suggested that genes for skin melanogenesis, hormone regulation, angiogenesis, vasodilation, mitochondrial respiration, and immune regulation were under positive selection in a study on the genetic adaptation of domestic chickens to tropical climate [5]. In a study on the adaptation of local Chinese domestic chickens to hot, arid and harsh environments, it was revealed that genes related to circulatory and vascular development, neural development, apoptosis, stress response, and fatty acid metabolism were selected [69]. In 2022, Zhao et al. found that genes for metabolism, immunity, hair follicle development, blood pressure regulation, and angiogenesis were associated with the cold adaptation of Chinese local chickens [6].

We identified genes that are closely related to the regulation of climate adaptation within the selected regions of cold adaptation in Huoyan geese, including metabolism-related, angiogenesis and contraction-related, circulatory system-related, apoptosis-related, immune-related, stress-related, and neuron-related genes. Metabolism-related strongly selected genes included *PIP5K1B* and *NMNAT3*. *PIP5K1B* encodes phosphatidylinositol 4-phosphate 5-kinase β type I (PIP5K1β), a member of the PIP5K family, and the other two isoforms are PIP5K1α and PIP5K1γ [70]. The gene generates 4,5-phosphatidylinositol-4,5-bisphosphate (PI(4,5)P2) by phosphorylating phosphatidylinositol 4-phosphate (PI(4)P) and has been shown to play a regulatory role in apoptosis [71,72]. For animals living in cold regions, the low-temperature environment leads to cold-adapted reactions, which include those affecting apoptosis [73,74,75]. There are a number of studies that have shown that phospholipid-based signaling plays an important role in response to cold exposure [76]. PI(4,5)P2 levels change as one of the early responses to cold exposure [77]. In the yeast *Saccharomyces cerevisiae*, it has been shown that PI(4,5)P2 levels in the cytoplasmic membrane decrease with a decrease in temperature and are involved in the regulation of lipid homeostasis [78]. In plants, PI(4,5)P2 and its derivative PI(1,4,5)P3 accumulate in cold-stressed plants [79]. Therefore, we suggested that the cold adaptation of Chinese local geese of Northern and Southern China are probably related to the regulation of PI(4,5)P2 levels by *PIP5K1B*.

NMNAT3, encoded by *NMNAT3*, is a central enzyme in nicotinamide adenine dinucleotide (NAD+) biosynthesis and mainly catalyzes its de novo synthesis and recycling pathway [80]. NAD+ plays an important role in glycolysis, β-oxidation, and the tricarboxylic acid (TCA) cycle, and is associated with immunity and DNA repair [81]. Its metabolic disorders have been described as a variety of metabolic disease markers, including type 2 diabetes [82]. It has been shown that *NMNAT3* overexpression contributed to increased mitochondrial NAD+ levels and stimulated mitochondrial metabolism [83]. In mammals, *NMNAT3* is important for increasing metabolic levels and improving metabolic health [84]. Therefore, we suggested that cold adaptation in Chinese local geese probably caused *NMNAT3* under selection, which led to affect the energy metabolism of geese.

We identified three genes associated with heat adaptation among nine heat-adapted shared candidate genes: the neuron-related *AGTPBP1*, the metabolism-related *GDA*, and the apoptosis-related *NAA35*. Particularly, *GDA* is a metal-dependent hydrolase that catalyzes the hydrolysis of guanine to produce xanthine and ammonia [85]. *GDA* can regulate the level of intracellular purine derivatives and participate in multiple signaling pathways [86]. *GDA* can promote dendritic formation and regulate neuronal development in neurons [87,88]. Several studies have shown that *GDA* is associated with skin diseases and epidermal pigmentation. In seborrheic keratosis, guanine deaminase upregulation increased UV-induced keratinocyte senescence, via uric acid formation mediated by reactive oxygen species followed by DNA damage [89]. Melasma is a hyperpigmentation disorder caused by UV irradiation and inflammation [90], and the expression of *GDA* mRNA is 5~14-fold higher in melasma lesions than in non-lesioned tissues [91]. In 2020, Jung et al. found that *GDA* in epidermal keratinocytes may promote melanogenesis by upregulating SCF and ET-1 [92]. Therefore, we suggested that the heat adaptation of Southern Chinese local geese may be related to skin pigmentation, nervous system, and apoptosis.

## 5. Conclusions

In this study, a joint statistical analysis was used to identify the genetic mechanisms underlying the specific phenotypes and climatic adaptations of Huoyan geese. Our results indicated that the upper eyelid coloboma trait in Huoyan geese was probably generated by the effect of the *PTPRM* gene located on the Z chromosome. *TYRP1* is a possible candidate gene for the Huoyan gosling feather color trait. In addition, we performed a selective sweep analysis of Huoyan geese and Southern Chinese local geese, and we obtained a large number of genes related to cold adaptation, mainly involved in physiological functions such as metabolism, angiogenesis and contraction, circulatory system, apoptosis, immunity, stress, and neural system. The most interesting candidates for cold adaptation are *PIP5K1B* and *NMNAT3* that are associated with energy metabolism and stress. The most interesting candidates for heat adaptation are *AGTPBP1,* associated with neurology; *GDA,* associated with skin pigmentation; and *NAA35,* associated with apoptosis. Our study is the first to reveal the genetic mechanisms underlying the specific phenotypes and climate adaptation of Chinese local geese, providing the theoretical basis for the subsequent research.

## Data Availability

Whole-genome resequencing analysis of 72 Chinese domestic geese is available on NCBI (BioProject ID: PRJNA989585).

## Supplementary Materials

The following supporting information can be downloaded at: https://www.mdpi.com/article/10.3390/ani13233608/s1, Figure S1: Male/Female coverage ratios for *TYRP1* and *EDNRB2*; Figure S2: Functional enrichment analysis of the cold adaptation of Huoyan geese; Table S1: Categories of goose breeds; Table S2: Summary of genome sequencing and mapping statistic; Table S3: Results of cross-validation error values for population structure analysis; Table S4: Shared candidate genes for the eyelid coloboma traits in Huoyan goose; Table S5: Shared candidate genes for feather color traits in 1-day-old Huoyan goose; Table S6: Shared candidate genes for the cold adaptation of Huoyan geese; Table S7: Shared candidate genes for the heat adaptation of Southern Chinese local geese; Table S8: GO analysis of the shared candidate genes for the cold adaptation of Huoyan geese.
