# Supplementary material for: Genomic Analysis Reveals Candidate Genes Underlying Sex-Linked Eyelid Coloboma, Feather Color Traits, and Climatic Adaptation in Huoyan Geese
Source: Animals (Basel). 2023 Nov 22;13(23):3608. doi: 10.3390/ani13233608 (PMC10705202; doi:10.3390/ani13233608)
Supplement: Supplementary file 1 [file animals-13-03608-s001.zip › Supplementary Figure.pdf]

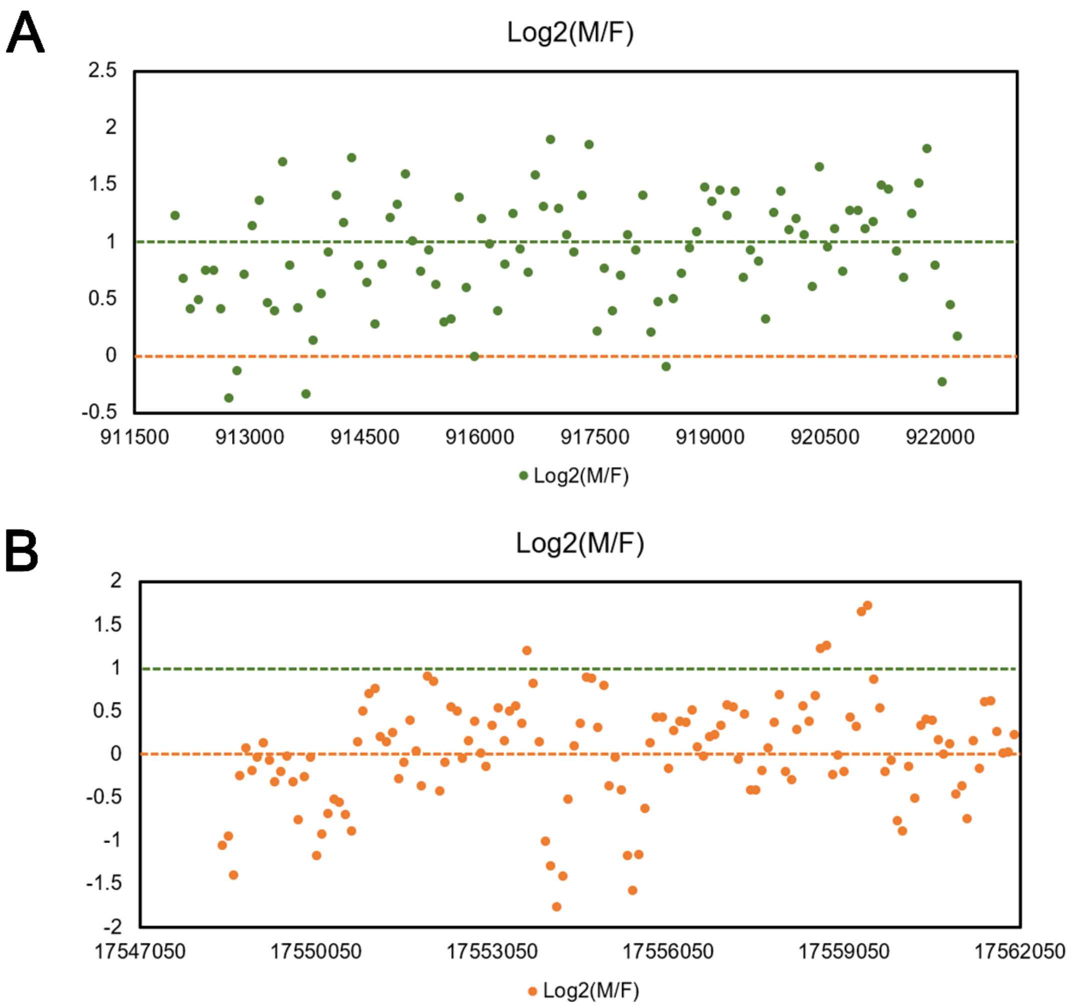

Figure S1 Male:female coverage ratios for (A) *TYRP1* and (B) *EDNRB2*, plotted by gene length. Each point represents a 100bp window, the orange line is the theoretical expectation for autosomes (log2 (M/F coverage) = 0), the green line is the theoretical expectation for Z chromosome (log2 (M/F coverage) = 1).

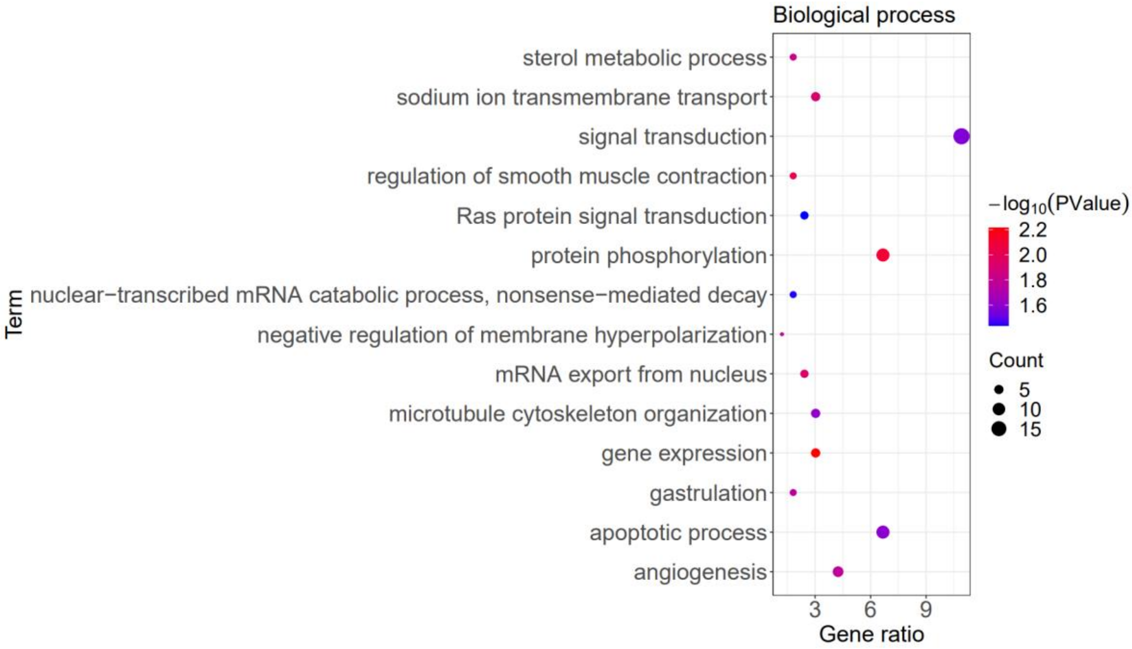

Figure S2 Functional enrichment analysis of cold adaptation of Huoyan geese. The terms are biological processes.
